# Integrative Evidence on Mulberry Extract for Modulating Metabolic Risk Factors Associated with Vascular Dementia

**DOI:** 10.3390/ijms26178380

**Published:** 2025-08-28

**Authors:** Jui-Ting Yu, Chen-Pi Li, Yao Hsiao, Kuan-Po Cheng, Ru-Yin Tsai

**Affiliations:** 1 Division of Hematology/Medical Oncology, Department of Medicine, Tung’s Taichung MetroHarbor Hospital, Taichung 43503, Taiwan; braves0818@gmail.com; 2 Department of Nursing, Tung’s Taichung MetroHarbor Hospital, Taichung 43503, Taiwan; t8369@ms.sltung.com.tw; 3 Department of Nursing, National Taichung University of Science and Technology, Taichung 40343, Taiwan; 4 School of Medicine, Chung Shan Medical University, Taichung 40201, Taiwan; s1001085@gm.csmu.edu.tw (Y.H.); taiwantaiwan73@gmail.com (K.-P.C.); 5 Department of Anatomy, School of Medicine, Chung Shan Medical University, Taichung 40201, Taiwan; 6 Department of Medical Education, Chung Shan Medical University Hospital, Taichung 40201, Taiwan

**Keywords:** inflammation, neurocognitive disorder, lipid profile, body mass index, cytokine, glucose metabolism

## Abstract

Metabolic syndrome refers to a group of conditions that commonly occur together, including abdominal obesity, high blood pressure, elevated blood sugar, high triglyceride levels, and low high-density lipoprotein cholesterol (HDL). These factors collectively increase the risk of cardiovascular disease, diabetes, and cognitive impairment. Recent research has identified a connection between metabolic syndrome and cognitive disorders such as mild cognitive impairment and vascular dementia (VaD). Mulberry (*Morus alba* L.) is a natural source of bioactive compounds with antioxidant, anti-inflammatory, and lipid-regulating properties. This meta-analysis assessed the potential of mulberry extract as an adjunctive treatment for metabolic risk factors linked to vascular dementia. We systematically reviewed randomized controlled trials (RCTs) published up to May 2025 that compared mulberry extract to placebo or standard care in adults with metabolic disorders. Fifteen trials including 1202 participants met the inclusion criteria. The primary outcomes were fasting glucose, fasting insulin, liver enzyme levels, lipid profiles, and inflammatory markers such as tumor necrosis factor-alpha (TNF-α), interleukin-6 (IL-6), and high-sensitivity C-reactive protein (hs-CRP). The pooled results indicated that mulberry supplementation improved blood sugar control and lowered total cholesterol, low-density lipoprotein cholesterol (LDL), triglycerides, fasting blood glucose, glycosylated hemoglobin (HbA1c), homeostasis model assessment for insulin resistance (HOMA-IR), and inflammatory markers. Aspartate aminotransferase (AST) improved, whereas alanine aminotransferase (ALT) showed no significant change. Subgroup analyses revealed that greater benefits were associated with shorter treatment durations and doses below 500 milligrams per day. Furthermore, extracts from different parts of the mulberry plant showed varying effects on lipid and glucose metabolism. None of the included trials directly measured cognitive or neurovascular outcomes, so any potential neurovascular protection is inferred from changes in metabolic and inflammatory markers rather than demonstrated. In summary, these findings suggest that mulberry extract may be a promising complementary approach for managing metabolic risk factors in people at risk for VaD. However, further large-scale and rigorously designed studies are required to confirm its clinical benefits and to identify the most effective preparations.

## 1. Introduction

Metabolic syndrome represents a growing global health challenge, defined by the presence of interrelated risk factors such as central obesity, hypertension, hyperglycemia, dyslipidemia, and reduced HDL [1]. Individuals with metabolic syndrome are at greater risk for developing cardiovascular disease, type 2 diabetes (T2DM), and other complications that reduce both lifespan and quality of life [2].

Recent studies have revealed that metabolic syndrome also contributes to neurodegenerative processes and cognitive impairment [1,3]. The aggregation of metabolic risk factors can accelerate VaD and promote chronic low-grade inflammation, which are implicated in the development and progression of dementia, especially vascular dementia [1,3]. Vascular dementia is recognized as the second most common cause of dementia after Alzheimer’s disease and is closely related to cerebrovascular pathology and the presence of metabolic disorders [4,5]. Given the rising prevalence of both metabolic syndrome and dementia in aging populations, there is a critical need to identify safe and effective strategies for risk reduction and prevention.

Mulberry (*Morus alba* L.), a plant widely cultivated in Asia, has been traditionally used in Chinese and Korean medicine to manage blood sugar and cholesterol levels [6]. Both its leaves and fruits contain bioactive compounds, including flavonoids, anthocyanins, and 1-deoxynojirimycin (DNJ), which have demonstrated antioxidant, anti-inflammatory, and glucose-lowering properties [7,8]. In animal and in vitro studies, mulberry extracts have been shown to inhibit α-glucosidase activity, reduce lipid accumulation, and suppress inflammatory cytokines such as TNF-α and IL-6 [9,10], all of which are important targets in the management of metabolic syndrome and its neurological consequences.

Although several reviews have discussed the general health benefits of mulberry, its potential role as a complementary therapy for metabolic risk factors associated with VaD has not been fully clarified. To address this gap, the present meta-analysis systematically evaluates the VaD from RCTs investigating mulberry extract supplementation in adults with metabolic disorders, with a focus on outcomes relevant to metabolic risk factors.

## 2. Results

### 2.1. Study Search and Characteristics of Included Patients

**Figure 1:**
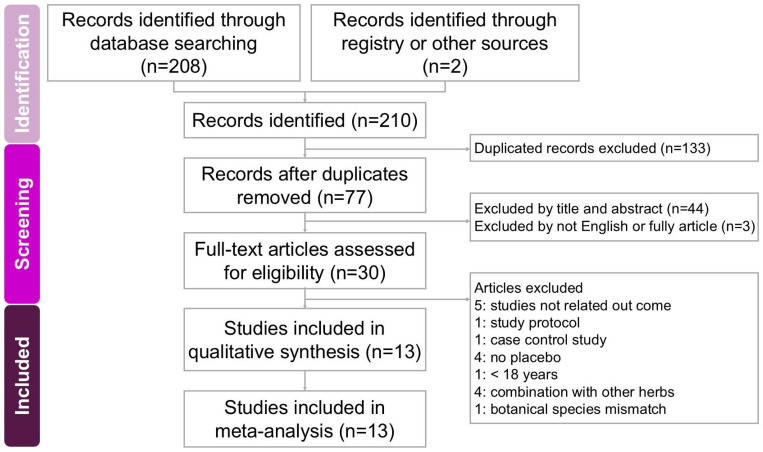
Flowchart showing the study selection process for this systematic review and meta-analysis on the effects of mulberry extract interventions for improving metabolic risk factors. From an initial 210 records identified, 13 studies met the inclusion criteria and were included in the final analysis [10,28,29,30,31,32,33,34,35,36,37,38,39].

The process of identifying and selecting trials for inclusion is summarized in Figure 1. We searched four databases, including PubMed, Embase, Web of Science, and the Cochrane Library, and we also screened the PubMed “Related Articles” feature, which yielded 210 records in total. After removing duplicates, 77 unique studies remained for initial screening based on titles and abstracts, leading to the exclusion of 47 studies that did not meet the inclusion criteria. The full texts of the remaining 30 articles were then reviewed in detail, resulting in the exclusion of 17 additional studies. Reasons for exclusion included studies enrolling participants under 18 years of age [11], protocol reports without outcome data [12], trials evaluating outcomes unrelated to the objectives of this analysis [13,14,15,16,17], botanical species mismatch [18], one study using a case-control design [19], studies combining mulberry with other herbs [20,21,22,23], and studies lacking a placebo-controlled design [24,25,26,27]. In total, 13 RCTs were included in the final quantitative synthesis [10,28,29,30,31,32,33,34,35,36,37,38,39]. All selected studies were published in English. The main characteristics of these trials are presented in Table 1. The included studies, published between 2001 and 2024, enrolled a total of 1202 participants and assessed the effects of mulberry extract supplementation on glucose metabolism, lipid metabolism, and pro-inflammatory cytokines, among other related outcomes.

### 2.2. Quality Assessment

**Figure 2:**
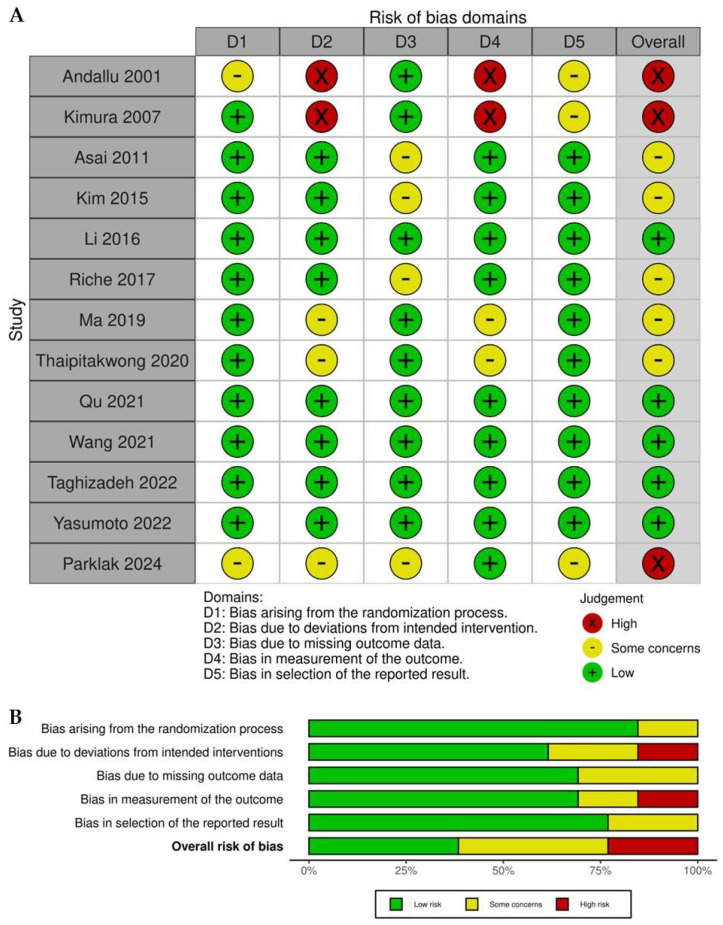
Assessment of methodological quality for the included trials [10,28,29,30,31,32,33,34,35,36,37,38,39]. Panel (A) shows the risk of bias evaluation for each individual study, conducted using the Risk of Bias 2.0 tool and visualized with the RoB visualization platform (https://mcguinlu.shinyapps.io/robvis/, accessed on 20 August 2025). Panel (B) provides a summary of the overall risk of bias distribution as percentages, reflecting both intention-to-treat and per-protocol analyses across the included studies.

The risk of bias in the included studies was evaluated using the Cochrane Risk of Bias 2.0 tool. Figure 2A presents the domain-level assessments for each study, while Figure 2B summarizes the overall distribution of risk levels. Of the thirteen studies included, five were rated as having a low overall risk of bias, five were classified as having some concerns, and three were judged to be at high risk. The domains most commonly associated with high risk were deviations from intended interventions and measurement of outcomes, particularly in the studies by Andallu [28], Kimura [29], and Parklak [10]. These studies contributed to the overall classification of high risk. The domains related to randomization and selection of reported results were most consistently rated as low risk. Similarly, bias due to missing outcome data was generally assessed as low risk or as presenting only some concerns. In summary, most studies demonstrated acceptable methodological quality; however, limitations related to blinding, adherence, or selective reporting were noted in certain studies and should be considered when interpreting the pooled results.

### 2.3. Impact of Mulberry Extract on Cholesterol

**Figure 3:**
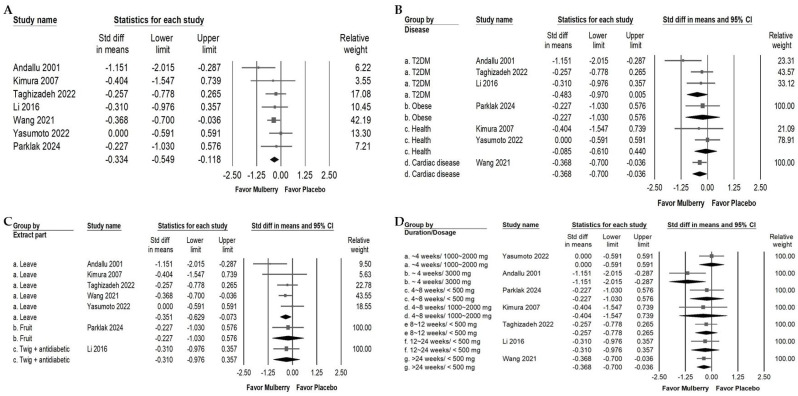
Forest plot summarizing the impact of mulberry supplementation on TC levels. Panel (A) presents the overall effect on TC. Elevated TC is a modifiable risk factor for atherosclerotic disease and is associated with vascular cognitive impairment. Panel (B) examines effects by diagnosis, Panel (C) evaluates outcomes by mulberry extraction part, and Panel (D) evaluates duration and dosage. Squares represent individual study effect estimates with 95% confidence intervals, while the diamonds at the bottom of each panel depict the pooled effect size, highlighting the overall impact on fasting blood glucose.

Mulberry extract supplementation was associated with a small reduction in TC (Figure 3A; overall random effect: −0.334, 95% CI: −0.549 to −0.118; *I*^2^ < 0.001%, *p* = 0.560). Subgroup analyses evaluating the participant health status (Figure 3B) revealed notably small TC reductions among cardiac disease individuals (overall random effect: −0.368; 95% CI: −0.700 to −0.036; *I*^2^ < 0.001%, *p* > 0.999). Conversely, no significant TC lowering effects were found in populations with type 2 diabetes mellitus (overall random effect: −0.483; 95% CI: −0.970 to 0.005; *I*^2^ = 37.922%, *p* = 0.200), in obese individuals (overall random effect: −0.227; 95% CI: −1.030 to 0.576; *I*^2^ < 0.001%, *p* > 0.999), or in healthy individuals (overall random effect: −0.085; 95% CI: −0.610 to 0.440; *I*^2^ < 0.001%, *p* > 0.999).

Figure 3C illustrates the effects of different parts of the mulberry plant used in the interventions. Mulberry leaf extract demonstrated a small reduction in TC, with an overall random effect of −0.351 (95% CI: −0.629 to −0.073; *I*^2^ = 16.496%, *p* = 0.310). In contrast, the mulberry fruit extract did not show a significant effect, with an overall random effect of −0.227 (95% CI: −1.030 to 0.576; *I*^2^ < 0.001%, *p* > 0.999). Interestingly, mulberry twig extract administered together with the usual antidiabetic medications used by patients did not result in a significant reduction in TC (overall random effect: −0.310; 95% CI: −0.976 to 0.357; *I*^2^ < 0.001%, *p* > 0.999); however, this finding is derived from a single RCT and should be interpreted with caution.

As shown in Figure 3D, subgroup analysis by intervention duration and dosage indicated that a large and significant reduction in TC occurred when the intervention lasted less than 4 weeks and the dosage exceeded 3000 mg per day (overall random effects model: −1.151; 95% CI: −2.015 to −0.287; *I*^2^ < 0.001%, *p* > 0.999). The data also indicate that interventions longer than 24 weeks with doses lower than 500 mg per day produced a small but significant additional benefit (overall random effects model: −0.368; 95% CI: −0.700 to −0.036; *I*^2^ < 0.001%, *p* > 0.999). No significant effects were found in other subgroups, including dosages between 1000 and 2000 mg per day under 4 weeks (overall random effect: −0.000; 95% CI: −0.591 to 0.591; *I*^2^ < 0.001%, *p* > 0.999), less than 500 mg per day for 4 to 8 weeks (overall random effect: −0.227; 95% CI: −1.030 to 0.576; *I*^2^ < 0.001%, *p* > 0.999), between 1000 and 2000 mg per day for 4 to 8 weeks (overall random effect: −0.404; 95% CI: −1.547 to 0.739; *I*^2^ < 0.001%, *p* > 0.999), less than 500 mg per day for 8 to 12 weeks (overall random effect: −0.257; 95% CI: −0.778 to 0.265; *I*^2^ < 0.001%, *p* > 0.999), and less than 500 mg per day for 12 to 24 weeks (overall random effect: −0.310; 95% CI: −0.976 to 0.357; *I*^2^ < 0.001%, *p* > 0.999). Taken together, these findings suggest that the effect of mulberry on total cholesterol may depend on intervention duration, participant characteristics, and the plant part used. Because several subgroups include only a single randomized controlled trial, these estimates should be interpreted with caution.

### 2.4. Influence of Mulberry Extract on TG, LDL, and HDL

**Figure 4:**
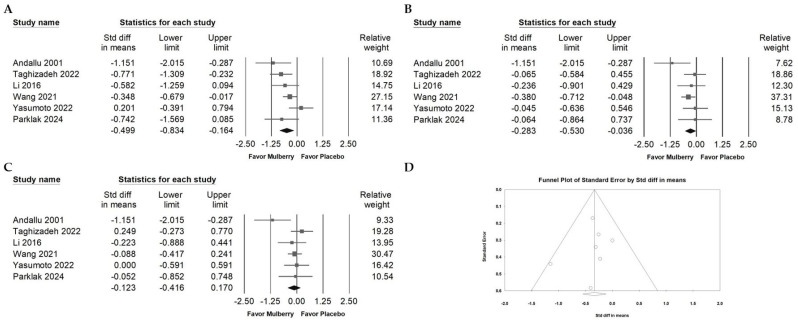
Forest plots of lipid outcomes with clinical relevance. Panel (A) shows changes in TG. Elevated TG is a component of atherogenic dyslipidemia and contributes to cardiometabolic risk. Panel (B) displays LDL. LDL is a causal risk factor for atherosclerotic disease and is associated with vascular cognitive impairment and vascular dementia. Panel (C) reports HDL. HDL is generally associated with lower cardiometabolic risk. Panel (D) presents a funnel plot for the LDL analysis to assess small-study effects. Lines represent confidence intervals around study level effect estimates. Each circle represents a study, with larger circles indicating greater weight or a larger sample size. The diamond shows the pooled effect, with its center marking the overall estimate and its width indicating the confidence interval.

Mulberry extract supplementation was associated with a small reduction in triglycerides (Figure 4A; overall random effect: −0.499, 95% CI: −0.834 to −0.164; *I*^2^ = 47.501%, *p* = 0.090) and in LDL cholesterol (Figure 4B; overall random effect: −0.283, 95% CI: −0.530 to −0.036; *I*^2^ = 13.984%, *p* = 0.325). No significant change was observed in HDL cholesterol (Figure 4C; overall random effect: −0.123, 95% CI: −0.416 to 0.170; *I*^2^ = 34.757%, *p* = 0.176). Taken together, these findings suggest that mulberry extract may modestly improve lipid metabolism by lowering LDL and triglycerides, while its influence on HDL appears limited.

Egger’s regression analysis showed no statistically significant evidence of publication bias among the included studies (*p* = 0.65638). Nevertheless, the funnel plot for total cholesterol (Figure 4D) appeared asymmetric upon visual inspection, suggesting a possible absence of smaller studies with null or positive findings. Although the statistical test did not detect bias, this pattern warrants cautious interpretation. To investigate further, a trim-and-fill analysis was performed. The adjusted effect size (point estimate: −0.33382; 95% CI: −0.59562 to −0.20301) showed only a very small difference from the original estimate (point estimate: −0.33382; 95% CI: −0.54931 to −0.11833), indicating that any potential publication bias likely had minimal influence on the overall results.

### 2.5. Impact of Mulberry Extract on Blood Fasting Glucose

**Figure 5:**
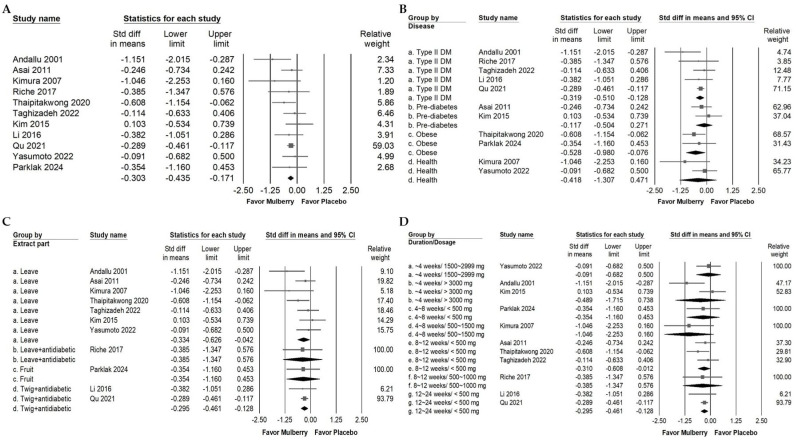
Forest plot summarizing the impact of mulberry extract supplementation on fasting blood glucose levels. Panel (A) presents the overall effect on fasting blood glucose. Elevated fasting blood glucose reflects impaired glycemic control and is associated with higher risks of type 2 diabetes, endothelial dysfunction, and vascular cognitive impairment. Panel (B) examines effects by diagnosis, Panel (C) evaluates outcomes by part of mulberry, and Panel (D) evaluates duration and dosage. Squares represent individual study effect estimates with 95% confidence intervals, while the diamonds at the bottom of each panel depict the pooled effect size, highlighting the overall impact on fasting blood glucose.

Figure 5A shows that all interventions lowered fasting blood glucose relative to placebo. The pooled random effects estimate indicated a small decrease (−0.303; 95% CI: −0.435 to −0.171; *I*^2^ < 0.001%, *p* = 0.523). Subgroup analyses by health status (Figure 5B) suggested a moderate reduction among individuals with obesity (overall random effect: −0.528; 95% CI: −0.980 to −0.076; *I*^2^ < 0.001%, *p* = 0.609) and a smaller reduction among patients with type 2 diabetes mellitus (overall random effect: −0.319; 95% CI: −0.510 to −0.128; *I*^2^ = 7.287%, *p* = 0.365). In contrast, participants with prediabetes showed no clear change (overall random effect: −0.117; 95% CI: −0.504 to 0.271; *I*^2^ < 0.001%, *p* = 0.395), and healthy individuals showed no meaningful reduction (overall random effect: −0.418; 95% CI: −1.307 to 0.471; *I*^2^ = 48.526%, *p* = 0.163).

Regarding plant part (Figure 5C), leaf preparations used alone showed a small reduction in fasting blood glucose (overall random effect: −0.334; 95% CI: −0.626 to −0.042; *I*^2^ = 33.120%, *p* = 0.175). Twig extracts given together with usual antidiabetic medications produced a smaller but statistically significant reduction (overall random effect: −0.295; 95% CI: −0.461 to −0.128; *I*^2^ < 0.001%, *p* = 0.791). In contrast, fruit extracts (overall random effect: −0.354; 95% CI: −1.160 to 0.453; *I*^2^ < 0.001%, *p* > 0.999) and leaf extracts administered with usual antidiabetic medications (overall random effect: −0.385; 95% CI: −1.347 to 0.576; *I*^2^ < 0.001%, *p* > 0.999) did not demonstrate a significant reduction in fasting glucose.

As shown in Figure 5D, a small reduction in fasting blood glucose was observed with interventions of 8 to 12 weeks at doses below 500 mg per day (random effects model: −0.310; 95% CI: −0.608 to −0.012; *I*^2^ < 0.001%, *p* = 0.450) and with 12 to 24 weeks at doses below 500 mg per day (random effects model: −0.295; 95% CI: −0.461 to −0.128; *I*^2^ < 0.001%, *p* = 0.791). Other subgroup categories did not show significant effects, including less than 4 weeks with doses of 1500 to 2999 mg per day (overall random effect: −0.091; 95% CI: −0.682 to 0.500; *I*^2^ < 0.001%, *p* > 0.999) and greater than 3000 mg per day (overall random effect: −0.489; 95% CI: −1.715 to 0.738; *I*^2^ = 80.927%, *p* = 0.022). Interventions using under 500 mg per day for 4 to 8 weeks (overall random effect: −0.354; 95% CI: −1.160 to 0.453; *I*^2^ < 0.001%, *p* > 0.999), 500 to 1500 mg per day for 4 to 8 weeks (overall random effect: −1.046; 95% CI: −2.253 to 0.160; *I*^2^ < 0.001%, *p* > 0.999), and 500 to 1500 mg per day for 8 to 12 weeks (overall random effect: −0.385; 95% CI: −1.347 to 0.576; *I*^2^ < 0.001%, *p* > 0.999) also showed no significant effect.

### 2.6. Effects of Mulberry Extract on Serum Insulin, HbA1c, HOMA-IR, and BW

**Figure 6:**
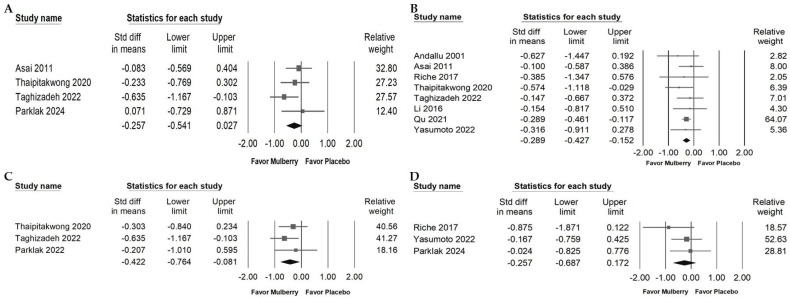
Forest plots of glycemic control markers with clinical relevance. Panel (A) shows fasting serum insulin. Elevated fasting insulin reflects compensatory hyperinsulinemia and insulin resistance. Panel (B) shows HbA1c levels. HbA1c captures average glycemia over approximately three months and is strongly linked to microvascular and macrovascular complications. Panel (C) shows HOMA-IR, an index of insulin resistance that correlates with cardiometabolic risk. Panel (D) shows body weight, included to contextualize whether glycemic effects occur independent of weight change. Squares indicate effect estimates with 95% confidence intervals, and diamonds at the bottom of each panel represent the overall pooled effect size, illustrating the intervention’s impact on glycemic markers.

The effects of mulberry extract on glycemic markers were evaluated across several endpoints. A slight reduction in fasting insulin was observed, but this change was not statistically significant (Figure 6A; random effects model: −0.257, 95% CI: −0.541 to 0.027; *I*^2^ = 2.655%, *p* = 0.379). In contrast, a small but significant decrease in HbA1c was detected, suggesting possible improvements in long-term glucose regulation (Figure 6B; random effects model: −0.289, 95% CI: −0.427 to −0.152; *I*^2^ < 0.001%, *p* = 0.905). Mulberry extract also led to a slight reduction in insulin resistance as indicated by HOMA IR (Figure 6C; random effects model: −0.442, 95% CI: −0.764 to −0.081; *I*^2^ < 0.001%, *p* = 0.583). No significant change was found in body weight (Figure 6D; random effects model: −0.257, 95% CI: −0.608 to 0.172; *I*^2^ < 0.001%, *p* = 0.389). Overall, these findings suggest that mulberry extract may support improvements in long-term glycemic control, while effects on fasting insulin and other metabolic outcomes remain uncertain and warrant further investigation.

### 2.7. Impact of Mulberry Extract on Liver Injury Markers and Blood Pressure

**Figure 7:**
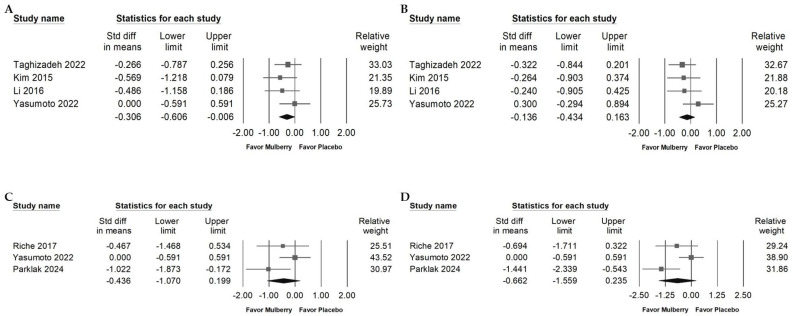
Forest plots of hepatic enzymes and blood pressure with clinical relevance. Panel (A) displays changes in AST levels. AST is present in liver, heart, and skeletal muscle, and elevations may reflect systemic cellular injury. Panel (B) shows ALT, a marker more specific to hepatocellular injury. Panel (C) shows systolic blood pressure (SBP) and Panel (D) shows diastolic blood pressure (DBP), both established risk factors for vascular and cognitive outcomes. Each square represents the standardized mean difference, with horizontal lines indicating the 95% confidence intervals. The diamond at the bottom of each panel represents the overall pooled effect size.

Mulberry extract supplementation was associated with a small reduction in AST (Figure 7A; overall random effect: −0.306, 95% CI: −0.6060 to −0.006; *I*^2^ < 0.001%, *p* = 0.150), while ALT showed no significant change (Figure 7B; overall random effect: −0.136, 95% CI: −0.434 to 0.163; *I*^2^ < 0.001%, *p* = 0.423). These findings suggest a possible hepatic benefit, particularly for AST. In contrast, there was no significant effect on blood pressure: systolic blood pressure (Figure 7C; overall random effect: −0.436, 95% CI: −1.070 to 0.199; *I*^2^ = 47.340%, *p* = 0.150) and diastolic blood pressure (Figure 7D; overall random effect: −0.662, 95% CI: −1.559 to 0.235; *I*^2^ = 71.843%, *p* = 0.029).

### 2.8. Effect of Mulberry Extract on Inflammatory Cytokines

**Figure 8:**
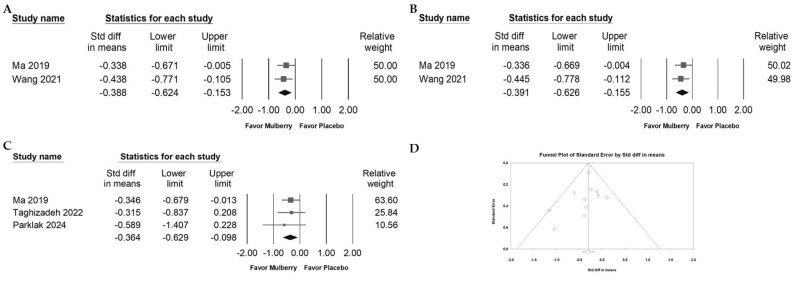
Forest plots of inflammatory markers with clinical relevance. Panel (A) shows TNF-α, a pro-inflammatory cytokine that promotes endothelial activation and may increase blood–brain barrier permeability. Panel (B) shows IL-6, which is associated with cardiometabolic and neurovascular risk. Panel (C) shows hs-CRP, a marker of systemic inflammation and cardiovascular risk. Squares represent effect estimates with 95 percent confidence intervals, and diamonds indicate the pooled effect size. Panel (D) presents a funnel plot for the studies in Figure 5A. Lines represent confidence intervals around effect estimates. Each circle represents a study, with larger circles indicating greater weight or larger sample size. The diamond shows the overall effect, with its center marking the pooled estimate and its width indicating the confidence interval.

Mulberry extract supplementation was associated with modest yet significant reductions in key pro-inflammatory cytokines. Specifically, meaningful decreases were found in TNF-α (Figure 8A; overall random effect: −0.388, 95% CI: −0.624 to −0.153; *I*^2^ < 0.001%, *p* = 0.676), IL-6 (Figure 8B; overall random effect: −0.391, 95% CI: −0.626 to −0.155; *I*^2^ < 0.001%, *p* = 0.652), and hs-CRP (Figure 8C; overall random effect: −0.364, 95% CI: −0.629 to −0.098; *I*^2^ < 0.001%, *p* = 0.845). These results suggest mulberry extract could potentially contribute to a reduction in systemic inflammatory markers.

### 2.9. Publication Bias of Included RCTs Reporting Fasting Blood Glucose Data

Egger’s regression for Figure 5A found no statistically significant evidence of publication bias among the included studies (*p* = 0.37171), consistent with general symmetry in the funnel plot (Figure 8D). Even so, visual inspection of the funnel plot for fasting blood glucose showed a sparsely populated lower right region, suggesting a possible absence of smaller studies with null or positive findings. Although the statistical test did not detect bias, this asymmetry warrants cautious interpretation. To examine this further, a trim-and-fill analysis was conducted. The adjusted effect size (point estimate: −0.30270; 95% CI: −0.43482 to −0.17058) was identical to the original estimate, indicating that any potential publication bias likely had minimal impact on the overall findings. Although statistical tests did not detect small-study effects, the observed funnel plot pattern is compatible with preferential nonpublication of small neutral trials in nutraceutical research. The unchanged trim-and-fill estimate suggests that any such bias likely had minimal impact on the pooled effect for fasting blood glucose. Even so, the certainty of evidence for this outcome should be interpreted with appropriate caution, and additional large, preregistered randomized trials would further clarify the magnitude of effect.

### 2.10. Safety and Tolerability

Across the 13 randomized trials, 1294 participants were randomized and 1202 were analyzed, with 92 withdrawals. Adverse event ascertainment and counts are summarized in Table S1. Mild gastrointestinal complaints were the most frequent events. No serious adverse events were judged related to mulberry-based interventions. Trials that monitored hypoglycemia reported no severe or clinically meaningful events. Overall, no major safety signal emerged and mulberry-based interventions were generally well tolerated (Table S1).

## 3. Discussion

This meta-analysis provides integrative evidence suggesting that mulberry (*Morus alba* L.) extract supplementation may be beneficial as an adjunctive strategy to address metabolic risk factors linked with vascular dementia. Mulberry extract significantly improved glycemic control and lipid metabolism, and it moderately reduced markers of inflammation and hepatic enzymes. Collectively, these improvements could potentially translate to neurovascular protective effects, as metabolic abnormalities and systemic inflammation are key drivers of cognitive decline and cerebrovascular dysfunction in vascular dementia patients.

Compared to a previous meta-analysis that concentrated on glucose regulation, lipid profiles, and blood pressure [40], the current study incorporated a greater number of RCTs, totaling fifteen, allowing for a more nuanced and robust evaluation. Our results indicate that mulberry extract supplementation contributes to a slight decrease in fasting blood glucose and produces small but statistically significant improvements in glycated hemoglobin and insulin resistance levels. These findings support the potential role of mulberry extract as a complementary strategy for improving long-term glycemic regulation, particularly in populations affected by dyslipidemia and obesity. The improvement observed in HbA1c is clinically meaningful, as this marker reflects average blood glucose levels over time and is strongly associated with vascular complications, including cerebral small vessel disease, which plays a critical role in the pathophysiology of vascular dementia [5,41,42]. Similarly, the observed reduction in HOMA-IR suggests that mulberry extract may modestly improve insulin sensitivity, which is relevant given that insulin resistance has been implicated in endothelial dysfunction and cerebral hypoperfusion [43], both of which contribute to cognitive decline in individuals with metabolic syndrome. Subgroup analysis revealed that the greatest reduction in fasting glucose occurred in studies with intervention durations of up to four weeks. In contrast, trials lasting more than twelve weeks demonstrated less pronounced effects. This temporal pattern is consistent with previous research on nutritional interventions [44], including those investigating vitamin D [45] and mineral supplementation [46], which found that the benefits of certain supplements may diminish over time. Possible explanations include physiological adaptation, metabolic compensation, or reduced adherence to supplementation protocols as the intervention period lengthens. These considerations are especially important in chronic disease management and prevention of neurovascular complications.

In terms of lipid metabolism, mulberry extract supplementation was associated with significant reductions in total cholesterol, LDL cholesterol, and triglyceride levels while having no meaningful impact on HDL cholesterol. These findings are consistent with previous evidence [40] linking the bioactive compounds in mulberry, such as flavonoids, alkaloids, and DNJ, to lipid-lowering mechanisms through modulation of hepatic cholesterol metabolism, inhibition of cholesterol synthesis, and enhanced bile acid excretion [26,27]. The observed improvements in total cholesterol and LDL are particularly relevant, as elevated levels of these lipids are established contributors to vascular dysfunction and cognitive impairment [1,43], underscoring the importance of lipid modulation in neurovascular protection [5]. The present analysis showed that mulberry extract supplementation did not lead to a significant increase in HDL cholesterol. This outcome may be attributed to the use of unsuitable dosages or inappropriate intervention durations, or to the limited capacity of bioactive compounds in mulberry to modulate HDL levels. Current evidence regarding the effects of plant-based interventions on HDL remains inconsistent. Some studies have reported that triglyceride levels may be reduced without corresponding changes in HDL [47,48], which could be explained by differences in the metabolic pathways regulating these lipid components [49]. However, the absence of a significant increase in HDL represents a limitation. The inability of mulberry to raise HDL levels suggests that while it may improve certain aspects of lipid metabolism, it may not achieve a fully balanced lipid profile when used alone. To address this limitation, future studies may investigate the combined use of mulberry with other bioactive agents, such as omega-3 fatty acids [50] or niacin [51], which are known to elevate HDL levels and may provide broader neurovascular protection.

The reduction in AST levels observed with mulberry extract supplementation, accompanied by only minimal changes in ALT, suggests a differential impact on hepatic enzymes. AST is distributed across various tissues including the liver, heart, and skeletal muscle, and its elevation often reflects generalized cellular injury [52]. In contrast, ALT is largely confined to liver cells and is considered a more specific biomarker for hepatocellular damage [53]. The selective decrease in AST may therefore indicate systemic biological effects of mulberry rather than liver-specific activity. This interpretation aligns with the antioxidant and anti-inflammatory properties of bioactive constituents in mulberry, such as flavonoids and DNJ, which are known to reduce oxidative stress, a key driver of elevated transaminase levels [26,54]. These findings point to a possible hepatoprotective effect of mulberry extract, potentially mediated through modulation of oxidative and inflammatory pathways that influence AST release. To further clarify this mechanism, future studies should incorporate tissue-specific biomarkers and explore dose–response effects. Consistent with previous research [40,55], our analysis also found no significant changes in body weight, systolic blood pressure, or diastolic blood pressure. This suggests that the metabolic improvements associated with mulberry supplementation are likely independent of changes in body mass or blood pressure regulation.

Inflammation is central to the pathology of VaD and related neurovascular disorders, promoting neuronal damage and vascular injury [1]. This meta-analysis demonstrates that mulberry supplementation has a significant anti-inflammatory effect, as shown by the consistent reductions in pro-inflammatory cytokines such as TNF-α, IL-6, and hs-CRP. Previous mechanistic studies have suggested that mulberry’s anti-inflammatory effects are mediated through the downregulation of nuclear factor kappa-B (NF-κB) signaling pathways, which may explain its broad systemic anti-inflammatory actions [56]. Inflammatory markers are central to the development and progression of chronic metabolic diseases, including non-alcoholic fatty liver disease and insulin resistance [57]. The reduction in TNF-α is particularly important, as this cytokine promotes the expression of cellular adhesion molecules, leading to damage of the basal membrane and increased permeability of the blood–brain barrier [58,59]. Inflammatory stimuli activate microglia, which subsequently release more pro-inflammatory cytokines such as IL-6 and TNF-α, contributing to neuronal damage. The observed reductions in IL-6 and hs-CRP further support the potential of mulberry extract to modulate systemic inflammation through its anti-inflammatory properties. These effects are likely mediated by the bioactive compounds found in mulberry, including flavonoids, alkaloids, and anthocyanins, which have been shown to inhibit key inflammatory pathways [60]. Consistent with our findings, a 24-week randomized, placebo-controlled trial in adults at increased risk for dementia showed that anthocyanin supplementation reduced C-reactive protein, lowered LDL cholesterol, and improved a composite cardiometabolic score versus placebo, with larger CRP reductions among participants with higher baseline inflammation [61]. The heterogeneity across included trials was generally low to moderate, suggesting a relatively consistent effect across different populations and study settings. However, variability in supplementation protocols—such as intervention duration, dosage, and plant part used—may have contributed to differences in treatment response. For example, some studies used only mulberry leaves, while others combined leaves with fruits or twigs, each of which contains distinct phytochemical profiles. The effectiveness of mulberry supplementation appeared to vary depending on the specific plant parts used in the intervention. This trend suggests that the therapeutic potential of mulberry may be enhanced through the synergistic action of its various bioactive compounds. For example, DNJ found in the leaves [28,29] and anthocyanins concentrated in the fruits [20,23] have both been shown to contribute to glycemic regulation and anti-inflammation defense. The presence of these compounds in combination may amplify metabolic and anti-inflammatory effects. These findings highlight the potential benefit of using whole-plant or multi-part formulations to more fully harness the therapeutic capacity of mulberry. Mulberry leaves contain quercetin, including quercetin 3-(6″)-malonylglucoside (Q3MG), which provides a plausible mechanistic link to the metabolic and anti-inflammatory pattern observed in this meta-analysis. Quercetin has been reported to improve metabolic dysfunction, reduce inflammation and oxidative stress, and modulate lipid handling in preclinical and clinical contexts [62,63,64]. Q3MG is a major quercetin glycoside in mulberry leaves and has been identified across cultivars, supporting its biological relevance in leaf-based preparations [65]. These data justify quantifying Q3MG and related conjugates in future trials and align with our finding that mulberry-based interventions exert small but consistent improvements in glycemic and lipid parameters.

This meta-analysis is subject to several limitations that should be acknowledged. Heterogeneity was low to moderate for several outcomes, but it was substantial for others, such as TG, HDL, HOMA IR, SBP, and DBP. These discrepancies may stem from differences in intervention protocols, including treatment duration, dosage, and characteristics of the study populations. Most included trials focused on individuals with metabolic disorders such as T2DM, obesity, or dyslipidemia, which limits the applicability of the findings to patients with vascular dementia. Although we prespecified subgroup analyses, several outcomes still exhibited substantial heterogeneity. Effects varied by baseline metabolic status. For total cholesterol, for example, we observed small changes in populations with cardiac disease and no clear effect in healthy individuals, those with obesity, or those with type 2 diabetes, as summarized in the subgroup figures (Figure 3B). Differences in extract preparation and plant parts also likely contributed to variability. Trials used leaves, twigs, or fruits in extract, powder, or tea forms with heterogeneous standardization, which complicates direct comparison across studies. Another important constraint is the relatively short duration of most interventions, with only a few studies lasting beyond twelve weeks. This restricts our understanding of whether the observed benefits are sustainable over time or if physiological adaptation may diminish their efficacy. Additionally, the diversity in mulberry formulations across studies complicates the identification of the most effective therapeutic composition. The interventions varied in terms of plant parts used, including leaves, fruits, and twigs, as well as in preparation forms such as extracts, powders, and teas. Although mulberry has a long history of safe use in traditional medicine, only a limited number of trials systematically evaluated adverse events or reported safety outcomes. This makes it challenging to determine a complete safety profile. Furthermore, lifestyle factors such as dietary habits and physical activity levels were not consistently controlled across studies, which may have introduced potential confounding effects. In addition, few trials uniformly controlled diet or physical activity, and some allowed stable background medications. These design features may have diluted or amplified the observed effects and should be considered when interpreting pooled estimates. While publication bias was not statistically evident based on Egger’s test and trim-and-fill analysis, reliance solely on published data raises concerns that studies with null or negative findings may be underrepresented. Lastly this synthesis did not include trials that directly assessed cognition, neuroimaging, cerebrovascular reactivity, or clinical neurovascular events. Therefore, references to possible neurovascular protection are indirect and rest on observed improvements in glycemic control, lipid fractions, liver enzymes, and inflammatory markers. These results should be viewed as hypothesis-generating for neurovascular outcomes, and future randomized trials should incorporate validated cognitive tests and vascular imaging to evaluate clinical relevance. Taken together, these limitations underscore the need for future well-conducted randomized controlled trials with longer follow-up periods, standardized formulations, robust safety assessments, and more diverse patient populations to better evaluate the clinical potential of mulberry in managing metabolic risk factors.

While this meta-analysis focused on systemic metabolic and inflammatory parameters, future trials should evaluate whether mulberry extract influences microvascular and neurovascular processes that contribute to cognitive decline. In vascular dementia and related conditions, cerebral small vessel disease, disruption of blood–brain barrier integrity, impaired neurovascular coupling, cerebral microhemorrhages, and endothelial dysfunction are central to pathogenesis [66]. Chronic metabolic dysregulation and low-grade inflammation can worsen barrier permeability, promote leukocyte trafficking, and blunt cerebral autoregulation, thereby accelerating neuronal injury [67,68]. Bioactive constituents of mulberry leaves and fruits, including quercetin glycosides, anthocyanins, and DNJ, have antioxidant and anti-inflammatory properties that may stabilize tight junction proteins in the blood–brain barrier, improve endothelial nitric oxide bioavailability, and preserve neurovascular coupling [63]. These hypotheses require targeted testing. Future randomized trials should prespecify neurovascular endpoints alongside metabolic outcomes. Suggested measures include dynamic-contrast-enhanced magnetic resonance imaging (DCE MRI) for blood–brain barrier (BBB) permeability, cerebrovascular reactivity to hypercapnia assessed by blood-oxygenation-level-dependent functional magnetic resonance imaging (BOLD fMRI) or transcranial Doppler ultrasound (TCD), task-based indices of neurovascular coupling, susceptibility-weighted imaging (SWI) for cerebral microbleeds, and retinal optical coherence tomography angiography (OCTA) as a microvascular proxy. Parallel assays of endothelial and barrier biomarkers in blood or cerebrospinal fluid, such as vascular cell adhesion molecule 1 (VCAM 1), matrix metalloproteinase 9 (MMP 9), S100 calcium binding protein B (S100B), and tight junction proteins including claudin 5, occludin, and zonula occludens 1 (ZO 1), together with cognitive tests of executive function and processing speed, would clarify whether systemic effects of mulberry supplementation are accompanied by measurable neurovascular change. Moreover, mulberry also contains prenylated flavones with preclinical geroscience signals. In model organisms, mulberrin (and the related morusin) extended lifespan in yeast and C. elegans via nutrient-sensing pathways, with supporting cell data showing reduced phosphorylation of p70S6K1 (Thr389) and increased autophagy [69]. These findings are hypothesis-generating and do not establish clinical anti-aging effects, but they provide a mechanistic rationale for targeted human studies.

## 4. Materials and Methods

### 4.1. Data Sources and Selection Criteria

A comprehensive search strategy was implemented to identify randomized controlled trials evaluating the effects of mulberry (*Morus alba* L.) extract on metabolic risk factors in populations at risk of vascular cognitive impairment or vascular dementia. Literature searches were conducted in four major electronic databases: PubMed, Embase, Cochrane Library, and Web of Science. The search included articles published up to May 2025 and was restricted to human studies and randomized controlled trials. The full PubMed search strategy was as follows: (“Morus alba” [Mesh] OR “mulberry” OR “mulberry extract” OR “mulberry leaf”) AND (“Metabolic Syndrome” [Mesh] OR “Dyslipidemias” [Mesh] OR “Insulin Resistance” [Mesh] OR “Blood Glucose” [Mesh] OR “Cholesterol” [Mesh] OR “Triglycerides” [Mesh] OR “Glycemic Control” OR “Lipid Profile” OR “Inflammation” OR “Vascular Dementia” OR “Cognitive Decline”). To ensure methodological rigor, study selection followed the Preferred Reporting Items for Systematic Reviews and Meta-Analyses (PRISMA) guidelines. In addition, reference lists of included studies were manually reviewed to identify any relevant publications not retrieved through database searches. Exclusion criteria included non-original studies such as case reports, conference abstracts, narrative reviews, editorials, letters, or studies involving animals or in vitro models. This systematic review and meta-analysis was prospectively registered in the PROSPERO database under the registration number CRD42024603080.

### 4.2. Selection of Studies

The screening and evaluation of studies were conducted independently by two researchers. In cases of disagreement or uncertainty, a third researcher was consulted to achieve consensus and ensure consistency throughout the selection process. Full-text versions of all potentially eligible articles were retrieved and examined in detail to confirm inclusion and extract relevant data. The overall study selection process is illustrated in the PRISMA flow diagram shown in Figure 1.

### 4.3. Data Extraction

Two researchers independently extracted data using a standardized template developed in accordance with the recommendations of the *Cochrane Handbook* [70]. Extracted information included study identifiers such as the authors’ names, year of publication, and country of origin, along with inclusion criteria for participants and key demographic characteristics such as sample size and age range. Additional data collected encompassed the study design, details of the intervention, outcomes assessed, and specific methodologies employed for outcome measurement.

### 4.4. Outcomes

The primary outcomes assessed in this study included fasting blood glucose, BW, and liver function markers such as AST and ALT. Lipid-related parameters, including TC, TG, HDL, and LDL, were also examined. Secondary outcomes consisted of metabolic indicators such as fasting plasma insulin, HbA1c, and the HOMA-IR. Additionally, inflammatory markers including TNF-*α*, hs-CRP, and IL-6, as well as systolic and diastolic blood pressure, were evaluated.

### 4.5. Assessment of Methodological Quality

The risk of bias for each included study was independently assessed by two researchers using the Cochrane Risk of Bias 2.0 (RoB 2) tool for the assessment. When discrepancies occurred, a third reviewer was consulted to facilitate discussion and reach a consensus. Studies were considered to have a high risk of bias if concerns were identified in one or more of the predefined assessment domains.

### 4.6. Data Analysis

Quantitative data synthesis was conducted by calculating the standardized mean difference along with the corresponding 95% confidence intervals (CIs) to compare outcomes between intervention and control groups. A random effects model was applied to account for variability across studies. All statistical analyses were performed using Comprehensive Meta-Analysis software, version 3 (Biostat, Englewood, NJ, USA). Heterogeneity was evaluated using the *I*^2^ statistic, with values greater than 50 percent interpreted as indicative of substantial heterogeneity. Publication bias was assessed through visual inspection of funnel plots and Egger’s regression test. A *p*-value less than 0.05 was considered statistically significant for most comparisons, while a threshold of 0.10 was used for evaluating potential publication bias. To explore possible sources of heterogeneity, subgroup analyses were performed. Sensitivity analyses were also conducted by systematically excluding individual studies to assess the robustness and reliability of the pooled results.

## 5. Conclusions

This meta-analysis underscores the potential of mulberry extract supplementation as a natural adjunctive approach for improving metabolic and inflammatory parameters related to vascular dementia. The findings support its adjunctive role in improving glycemic control, modulating lipid profiles, and attenuating systemic inflammation. However, further high-quality randomized controlled trials are needed to confirm these results and determine the clinical applicability of mulberry extract. Future research should focus on long-term outcomes, optimal dosing strategies and the comparative efficacy of different plant formulations across diverse populations.

## Figures and Tables

**Table 1:** Characteristics of included studies.

Author (Year)/Country	Diagnosis	Inclusion Criteria	Exclusion Criteria	Sample Size (% of Male)/Age	Study Design	Placebo Using	Intervention/Duration	Main Results	Secondary Results
~8 weeks									
Andallu (2001)/India [28]	Male type 2 diabetes patients	1. Age group: 40–60 years. 2. Fasting blood glucose > 150 mg/dL. 3. HbA1c > 12%.	Patients with severe complications or comorbidities.	P: 12 (100) I: 12 (100)/40 to 60 years.	RCT/not mentioned/placebo	Glibenclamide	Single *Morus indica* L. leaves/shade-dried, powdered, and provided in capsule form (3 g/day)/30 days	1. 27% reduction in fasting blood glucose (*p* < 0.01). 2. Reduced total cholesterol (12%), LDL (23%), triglycerides (16%), and VLDL (17%) significantly (*p* < 0.01).	1. Increased HDL by 18% (*p* < 0.01). 2. Significant reduction in plasma and erythrocyte membrane lipid peroxides.
Kimura (2007)/Japan [29]	Healthy volunteers	Aged around 25 years, with a BMI within the normal range.	Subjects with any underlying health conditions that could affect glucose metabolism or were currently taking any medication.	P: 6 I (DNJ 0.4 g): 6 I (DNJ 0.8 g): 6 I (DNJ 1.2 g): 6/average age of 25.3 years	RCT/not mentioned/placebo	0 g of DNJ	DNJ-enriched *Morus indica* L. leaf powder/38 days	At doses of 0.8 g and 1.2 g, mulberry significantly reduced postprandial blood glucose, and DNJ intake suppressed plasma insulin secretion. Lipid profiles remained unchanged.	No adverse reactions in either group.
Kim (2015)/Korea [39]	Prediabetic subjects	Fasting blood glucose levels between 100 and 125 mg/dL and normal HbA1c levels (<6.5%).	Severe heart, liver, or kidney conditions, pregnant or breastfeeding women, and those taking medications or supplements that may influence glucose or lipid metabolism.	P: 19 (47.3) I: 19 (31.5)/P: 50.16 ± 7.83 I: 53.00 ± 7.20	RCT/double-blind/placebo	Color-matched placebo (lactose tablets)	Single *Morus indica* L. leaf aqueous extract standardized to 3.6 mg/g of DNJ/4 weeks	Postprandial blood glucose significantly decreased at 30 and 60 min (*p* < 0.05), and insulin AUC was notably reduced after 4 weeks (*p* = 0.0207).	No significant changes in fasting blood glucose or HbA1c levels in either group.
Ma (2019)/China [32]	Stable angina pectoris in patients diagnosed with coronary heart disease and blood stasis syndrome	Aged 35–80 years with symptoms included chest pain, tightness, and shortness of breath.	Patients with stable angina pectoris caused by other heart diseases, a history of trauma or fever, severe heart failure (ejection fraction < 35%), malignant tumors, or other serious conditions were excluded. Pregnant or breastfeeding women were also not included.	P: 78 (53.8) I: 64 (68.7)/P: 68.16 ± 7.36 I: 65.42 ± 8.48	RCT/double-blind/placebo	Conventional treatment	Daily oral administration of DNJ (10 mg) extracted from *Morus indica* L. leaves/4 weeks	Reduced inflammatory markers such as hs-CRP, IL-6, TNF-α, and malondialdehyde (MDA), and increased antioxidant SOD levels (*p* < 0.05).	1. Increased left ventricular ejection fraction and reduced left ventricular mass index (*p* < 0.05). 2. Improved aortic elasticity and reduced atherosclerosis index (*p* < 0.05).
Yasumoto (2022)/Japan [38]	Healthy adults	Fasting glucose and 2 h post-OGTT in the normal or borderline range; able to attend visits; provided written informed consent.	On any medical treatment during the trial; systolic blood pressure < 90 mmHg; pregnancy or breastfeeding; recent blood donation beyond protocol limits; participation in other studies; cardiac, hepatic, or renal disorders, history of cardiac disease, diabetes mellitus, drug or food allergy, glaucoma, and hyponatremia.	P: 24 (50) I: 23 (52.2)/P: 46.4 ± 10.2 I: 43.3 ± 13.7	RCT/double-blind/placebo	Matching 500 mL green tea beverage without mulberry leaf extract, identical in appearance and flavor	Green tea beverage containing mulberry leaf extract 550 mg per 500 mL, one bottle with each meal, three times daily, for 4 weeks	Intervention group decreases in ALP and urea nitrogen and an increase in uric acid and a decrease in LDL cholesterol; at 4 weeks, higher uric acid and HDL cholesterol and lower LDL cholesterol, non-HDL cholesterol, and HbA1c.	No adverse reactions in either group.
Parklak (2024)/Thailand [10]	Adults with obesity and central obesity plus elevated blood pressure	BMI > 25 kg/m^2^; central obesity (waist > 90 cm men, >80 cm women); SBP > 130 mmHg and/or DBP > 85 mmHg; able to complete study procedures; provided consent.	Liver, kidney, heart, thyroid, or adrenal disease; cancer; antibiotics within 3 months; alcohol 2–3 times per week; established CVD, diabetes, or NAFLD; use of polyphenol-containing beverages or supplements.	*n*= 12 (58.3)/46.57 ± 8.49	RCT/single-blind/crossover study	Placebo beverage of identical volume and calories, without mulberry extract	Concentrated mulberry drink (CMD); total 100 g/day; duration 6 weeks	In this crossover trial, CMD intake lowered systolic and diastolic blood pressure (*p* < 0.05), reduced triglycerides compared with placebo (*p* < 0.05), and kept fasting plasma glucose stable while it rose during placebo (*p* < 0.05). CRP protein was also lower with CMD than with placebo (*p* < 0.05), whereas LDL and HDL did not change significantly.	Body composition (weight, BMI, fat mass, WC/HC) and heart rate showed no meaningful changes across phases.
12 weeks									
Asai (2011)/Japan [30]	Impaired glucose metabolism	Fasting plasma glucose between 100 and 140 mg/dL, indicating impaired glucose metabolism.	Severe medical illness, pregnancy, lactation, or those using any agent for blood glucose control were excluded.	P: 32 (68) I: 33 (63.6)/average age 53.5 ± 7.5 years	Crossover RCT/double-blind/placebo	Not mentioned	Study 1: single dose (DNJ); study 2: 12-week supplementation, 6 mg DNJ, three times daily	Significant improved postprandial glycemic control (*p* < 0.001).	No significant differences in fasting plasma glucose, HbA1c, or glycated albumin concentrations between groups.
Riche (2017)/United States [34]	Type 2 diabetes mellitus	Patients on monotherapy or oral combination therapy, with a stable hemoglobin A1C (7.0–8.0%) for at least 2 months.	Using insulin, alpha-glucosidase inhibitors, those with cardiovascular disease, hepatic or renal insufficiency, pregnant women, or those with non-compliance history.	P: 12 (42) I: 12 (42)/P: 56 ± 7.0 I: 57 ± 5.5	RCT/double-blind/placebo	Matching placebo capsules	Single *Morus indica* L. leaf extract, 1000 mg standardized, taken three times daily with meals/3 months	Significant reduction in postprandial blood glucose (*p* < 0.05).	No significant changes in body weight, blood pressure, or fasting glucose were observed between groups.
Thaipitakwong (2020)/Thailand [35]	Borderline diabetes in obese individuals	Obese individuals aged 20–65 years with fasting plasma glucose of 100–140 mg/dL or 2 h postprandial plasma glucose of 140–199 mg/dL.	Taking antihyperglycemic agents, severe complications such as renal or hepatic impairments, or pregnant and lactating women were excluded.	P: 26 (23) I: 28 (32.1)/P: 52.0 ± 8.22 I: 53.14 ± 5.48	RCT/controlled clinical trial	Maintained nutritional control only	*Mulberry* leaf powder containing 12 mg DNJ per dose, taken three times daily/12 weeks	1. Significant reduction in fasting plasma glucose (*p* = 0.002). 2. Significant reduction in HbA1c levels (*p* = 0.011).	1. Mild improvements in insulin resistance (HOMA-IR) with borderline significance (*p* = 0.057). 2. No changes in postprandial glucose or insulin levels.
Taghizadeh (2022)/Iran [36]	Type 2 diabetes mellitus	Aged 35–70 years with T2DM, diagnosed based on the American Diabetes Association criteria.	Taken mulberry extract within the last three months, those with changes in glucose-lowering medications, those using anticoagulants, pregnant or lactating women, or patients with malignancies and chronic liver diseases were excluded.	P: 28 (32.1) I: 29 (31.0)/P: 52.6 ± 6.95 I: 46.2 ± 20.1	RCT/double-blind/placebo	Matching placebo twice daily	*Morus alba* extract (300 mg) taken twice daily/12 weeks	1. Significant reduction in insulin levels (*p* = 0.026) and HOMA-IR (*p* = 0.02). 2. Significant increase in HDL (*p* = 0.001) and a reduction in malondialdehyde (MDA) levels (*p* < 0.0001). 3. No significant changes in fasting plasma glucose, triglycerides, or other lipid profiles between the groups.	No serious adverse effects were reported.
24 weeks									
Li (2016)/China [31]	Type 2 diabetes mellitus	Aged 18–70 years with T2DM (HbA1c between 7.0% and 10.0%) who were not using antidiabetic medications for at least 3 months before screening or had used antidiabetic medication for no more than 3 months in total.	Severe diabetes complications, gastrointestinal conditions, poor blood pressure control, and those with liver or kidney disease were excluded. Pregnant and lactating women were also excluded.	P: 15 (33.33) I: 23 (34.78)/P: 57 ± 6.70 I: 56 ± 9.71	RCT/double-blind/double-dummy/active-controlled, and multiple-dose clinical trial	Matching *mulberry twig alkaloid* and acarbose tablets	*Mulberry twig alkaloid* tablet, 50 mg three times daily, increased to 100 mg three times daily after 4 weeks/24 weeks	1. Significant reduction in HbA1c (*p* < 0.001). 2. 1 h and 2 h postprandial plasma glucose levels significantly decreased in both groups (*p* < 0.05).	No significant changes in fasting plasma glucose or lipid profiles in either group.
Qu (2021)/China [33]	Type 2 diabetes mellitus	Aged 18–70 years with T2DM (HbA1c between 7.0% and 10.0%) and fasting blood glucose less than 13 mmol/L. The patients had not received any antidiabetic therapy or had used it for less than 3 months.	Severe diabetic complications, liver or kidney dysfunction, cardiovascular diseases, gastrointestinal dysfunction, or those taking any medication affecting glucose metabolism were excluded. Pregnant women were also excluded.	P: 222 (53.2) I: 321 (48.6)/P: 54.2 ± 9.01 I: 54.9 ± 9.41	RCT/double-blind/double-dummy/active-controlled, and multiple-dose clinical trial	Matching *mulberry twig alkaloid* and acarbose tablets	Sangzhi alkaloids from mulberry twigs, 50 mg three times daily for the first 4 weeks, then 100 mg three times daily for 20 more weeks/24 weeks	HbA1c significantly reduced.	No significant differences in fasting blood glucose, 1 h or 2 h postprandial glucose levels between the two groups.
12 months									
Wang (2021)/China [37]	Coronary heart disease with atherosclerosis	Aged 40–80 years with coronary heart disease confirmed by coronary angiography, LDL cholesterol levels > 140 mg/dL, and a history of myocardial infarction or coronary artery interventions.	Severe hepatic or renal dysfunction, ongoing treatment with DNJ for more than one month, or allergic reactions to DNJ or the drugs used in the study. Pregnant or lactating women were also excluded.	P: 70 (20) I: 72 (27.7)/P: 57.45 ± 11.28 I: 58.93 ± 10.76	RCT/double-blind/placebo	Placebo made of starch was administered	*Mulberry leaf* extract (DNJ 150 mg/day in three doses)/1 year	1. Decreased inflammatory markers (TNF-α, IL-1β, IL-6) and increased IL-10 in the treatment group (*p* < 0.05). 2. Significant reductions in LDL-C and total cholesterol.	Significant reduction in carotid intima media thickness in the treatment group compared to the control group (*p* < 0.05).

AUC: area under the curve; BMI: body mass index; DNJ: 1-deoxynojirimycin; HbA1c: glycosylated hemoglobin; OGTT: oral glucose tolerance test; HDL: high-density lipoprotein cholesterol; HOMA-IR: homeostasis model assessment for insulin resistance; hs-CRP: high-sensitive C-reactive protein; LDL: low-density lipoprotein cholesterol; P: placebo; I: intervention.

## Data Availability

Data are included in the article/Supplementary Materials or are referenced in the article.

## Supplementary Materials

The following supporting information can be downloaded at: https://www.mdpi.com/article/10.3390/ijms26178380/s1. Reference [71] is cited in the Supplementary Materials.
