# Supplementary material for: Integrative Evidence on Mulberry Extract for Modulating Metabolic Risk Factors Associated with Vascular Dementia
Source: Int J Mol Sci. 2025 Aug 28;26(17):8380. doi: 10.3390/ijms26178380 (PMC12428632; doi:10.3390/ijms26178380)
Supplement: Supplementary file 1 [file ijms-26-08380-s001.zip › ijms-3829780-supplementary.pdf]

Table S1. Adverse events (AE) and safety outcomes in randomized controlled trials of mulberry derived interventions. [71]

Study	N randomized / N analyzed	AE collection and definition	Any AE n (%)	Serious AE	Discontinuation due to AE	Hypoglycemia events	Authors' safety conclusion
Andallu 2001	24 / 24 (12 per arm)	Not reported (NR)	0	NR	0	0	NR
Kimura 2007	24 / 24 (6 per arm)	NR	0	NR	0	0	DNJ-enriched powder did not cause hypoglycemia; short-term intake considered safe.
Asai 2011	76 / 65 (cross-over study)	AEs monitored during 12-week supplementation.	10.5	NR	0 (8 withdrew for personal reasons)	NR	No serious AEs; generally well tolerated
Kim 2015	42 / 38 (19 per arm)	gastrointestinal symptoms and safety labs assessed	9.5	NR	0 (4 withdrew for personal reasons/eligibility issues)	NR	Well tolerated; gastrointestinal adverse effects not significant
Li 2016	38 / 38 (intervention 23, placebo 15)	AEs recorded at each follow-up; prespecified definitions incl. hypoglycemia	0	NR	0	NR	NR
Riche 2017	24 / 17	NR	25	Hyperparathyroidism; none related to mulberry	6 (gastrointestinal symptoms most common).	No severe/ symptomatic hypoglycemia	No significant differences in most safety markers.
Ma 2019	144 / NR	NR	NR	NR	NR	NR	NR
Thaipitakwong 2020	54 / 54 (28 intervention, 26 placebo)	NR	0	NR	0	No signs or symptoms of hypoglycemia during 12 weeks.	Relatively safe and well tolerated; gastrointestinal symptoms common but declined over time; no hepatic/renal alterations.

Qu 2021	600 / 543 (321 intervention, 222 placebo)	NR	9	NR	57 (not reported)	None in either group.	Good safety.
Wang 2021	160 / 142 (72 intervention, 70 placebo)	Any side effect is a reasonable possibility that the medicine caused the adverse event.	11.2	NR	18 (not fully enumerated)	NR	Mulberry did not increase side effect.
Taghizadeh 2022	60 / 57 (29 intervention, 28 placebo)	NR	0.05	NR	3 (not fully enumerated)	NR	NR
Yasumoto 2022	48 / 44 (22 intervention, 22 placebo)	Primary endpoint: adverse reactions; Secondary: adverse events.	8.3	NR	4 (stomach).	NR	No adverse reactions observed; mulberry beverage safe for healthy adults
Parklak 2024	12/ 12 (cross-over study)	NR	0	NR	NR	NR	Authors note absence of side effects over 6 weeks.

71. Page, M. J.; McKenzie, J. E.; Bossuyt, P. M.; Boutron, I.; Hoffmann, T. C.; Mulrow, C. D.; Shamseer, L.; Tetzlaff, J. M.; Akl, E. A.; Brennan, S. E.; et al,. The PRISMA 2020 statement: an updated guideline for reporting systematic reviews. *BMJ* **2021,** 372, n71. doi: 10.1136/bmj.n71.
